# Alcohol consumption and future hospital usage: The EPIC-Norfolk prospective population study

**DOI:** 10.1371/journal.pone.0200747

**Published:** 2018-07-18

**Authors:** Robert Luben, Shabina Hayat, Angela Mulligan, Marleen Lentjes, Nicholas Wareham, Paul Pharoah, Kay-Tee Khaw

**Affiliations:** 1 Department of Public Health and Primary Care, Institute of Public Health, University of Cambridge, Cambridge, United Kingdom; 2 MRC Epidemiology Unit, University of Cambridge School of Clinical Medicine, Cambridge United Kingdom; CUNY, UNITED STATES

## Abstract

**Background:**

Heavy drinkers of alcohol are reported to use hospitals more than non-drinkers, but it is unclear whether light-to-moderate drinkers use hospitals more than non-drinkers.

**Objective:**

We examined the relationship between alcohol consumption in 10,883 men and 12,857 women aged 40–79 years in the general population and subsequent admissions to hospital and time spent in hospital.

**Methods:**

Participants from the EPIC-Norfolk prospective population-based study were followed for ten years (1999–2009) using record linkage.

**Results:**

Compared to current non-drinkers, men who reported any alcohol drinking had a lower risk of spending more than twenty days in hospital multivariable adjusted OR 0.80 (95%CI 0.68–0.94) after adjusting for age, smoking status, education, social class, body mass index and prevalent diseases. Women who were current drinkers were less likely to have any hospital admissions multivariable adjusted OR 0.84 (95%CI 0.74–0.95), seven or more admissions OR 0.77 (95% CI 0.66–0.88) or more than twenty hospital days OR 0.70 (95%CI 0.62–0.80). However, compared to lifelong abstainers, men who were former drinkers had higher risk of any hospital admissions multivariable adjusted OR 2.22 (95%CI 1.51–3.28) and women former drinkers had higher risk of seven or more admissions OR 1.30 (95%CI 1.01–1.67).

**Conclusion:**

Current alcohol consumption was associated with lower risk of future hospital usage compared with non-drinkers in this middle aged and older population. In men, this association may in part be due to whether former drinkers are included in the non-drinker reference group but in women, the association was consistent irrespective of the choice of reference group. In addition, there were few participants in this cohort with very high current alcohol intake. The measurement of past drinking, the separation of non-drinkers into former drinkers and lifelong abstainers and the choice of reference group are all influential in interpreting the risk of alcohol consumption on future hospitalisation.

## Introduction

Alcohol misuse and its consequences continue to have a profound effect on society in general and on health services in particular. In 2015 there were 8,758 alcohol-related deaths in the UK [1] but a much higher estimate of 21,162 deaths and 914,929 hospital admissions wholly or partly attributable to harm from alcohol in England in 2010/11 has been calculated [2]. The direct and indirect costs to the NHS attributable to alcohol misuse have been estimated at approximately 3.5 billion pounds every year with estimates placing the overall economic burden to be between 1.3% and 2.7% of UK annual GDP [3,4]. Alcohol has been linked to 230 disease and injury categories in systematic reviews and for the majority of these, higher consumption is associated with a greater likelihood of disease. However, the level and pattern of alcohol drinking that constitutes misuse or excess varies by condition. National drinking guidelines also vary widely [5–7], suggesting lack of agreement of the levels of consumption considered acceptable. Alcohol-attributable fractions (AAF), the proportion of a disease or outcome that is attributed to excess alcohol consumption, vary greatly by condition [8]. Liver disease for example, constitutes the third commonest cause of premature death in the UK and three-quarters of deaths from liver disease are the result of excess alcohol consumption [9]. Alcoholic beverages were classified as carcinogenic by the International Agency for Research on Cancer (IARC) and many cancers are partly attributable to alcohol with monotonic increasing risk albeit with AAF at much lower levels.

The relationship between alcohol consumption and future hospital usage at lower levels of consumption are less clear. Whether alcohol has a cardioprotective effect has been the subject of considerable debate over many years [8,10–14]. A large body of epidemiological evidence together with evidence for plausible biological mechanisms, have reported beneficial associations for ischaemic heart disease (IHD) and diabetes at moderate levels of alcohol intake. Associations with other diseases such as Alzheimer's disease and gall bladder disease have also been reported to be mainly beneficial in systematic reviews [8,15–21].

The UK Health Education Council's guidance on alcohol drinking limits, first introduced in 1984, suggested limits considerably higher than those now recommended [5]. Recent public health guidelines in the UK examining lifetime risk associated with alcohol intake recommended a maximum weekly consumption of 14 units or 112 grams (1 UK unit = 8 grams of alcohol) for both men and women. This is based on modelling of the chronic and acute effects of alcohol using published systematic reviews and meta-analysis as the evidence base [6]. However, drinking guidelines vary widely by country, and while this may reflect cultural norms it also suggests a lack of agreement of the level at which consumption becomes harmful [7].

We have previously reported that age, body mass index (BMI) and smoking status predict future hospital use in a community based population of middle aged and older men and women over a ten year period of follow-up [22]. In the analyses presented here, we examined the relationship between current alcohol consumption in this cohort and their subsequent hospital usage over a period of ten years. This paper examines whether current drinking behaviour predicts the frequency or total days of future hospital admission from any cause over a fixed ten year period. Though we did not aim to describe the numerous pathological mechanisms that might be involved, we explored how conditions commonly found in older people might influence the overall relationship between alcohol consumption and future hospital usage. Our study is not designed to derive a prognostic model for predicting hospital use but rather to examine the relationship between usual alcohol consumption patterns at the more moderate levels generally observed in middle aged and older men and women living in the community and subsequent hospital usage.

## Materials and methods

The European Prospective Investigation into Cancer in Norfolk (EPIC-Norfolk) is a general population cohort of men and women aged 40–79 years living in Norfolk recruited from general practices between 1993–1997. The National Health Service means that general practice registers approximate population registers. The study has ethics committee approval from Norfolk Research Ethics Committee (Rec Ref: 98CN01) and all participants gave informed signed consent for study participation including access to medical records. The methods used were carried out in accordance with the relevant guidelines and regulations.

The design and recruitment of the study has been described in detail elsewhere [23,24]. Briefly, 77,630 were invited to participate of whom 30,445 consented to take part and completed a lifestyle questionnaire and 25,639 men and women subsequently attended a health examination.

### Alcohol exposure definitions

In the baseline questionnaire, participants were asked “Are you a non-drinker/teetotaller now?” and “At present, about how many alcoholic drinks do you have each week” for four types of alcohol: Beer, cider or lager (pints); wine (glasses), sherry or fortified wines (glasses) and spirits (singles). Current non-drinkers were defined as those who answered “yes” to being a non-drinker now and did not report consuming beer, wine or spirits at present. Similarly, current drinkers were defined as answering “no” to the question or report drinking at present.

Participants were also asked “Have you ever drunk alcohol in the past?” and two similar questions relating to consumption of the four alcohol types when aged 20 and aged 30. Former drinkers were defined as current non-drinkers who answered “yes” to ever drinking alcohol or reported consuming alcohol aged 20 or 30. Lifelong abstainers were defined as participants who are neither current drinkers nor former drinkers.

Current units and past units were calculated from the questionnaire responses with one unit equal to a half pint of beer, one glass of wine or fortified wine or a single measure of spirits. The capacity of a glass was not specified, but assumed to be 125ml for wine and 50ml for fortified wines. An additional category “occasional”, representing consumption of less than one drink per week, contributed half a unit when ticked for an alcohol type. Heavy current drinkers are defined as participants with >35 current units per week while heavy former drinkers are defined as participants with >35 past units per week. Those with current units greater than zero were divided into four categories: (0,7], (7,14], (14,21] and >21 units per week. Past alcohol consumption is defined as the higher of units reported consumed aged 20 and aged 30.

### Other covariates

Participants were also asked details of their current job and their partner's current job. Occupational social class was defined according to the Registrar General's classification [25]. Non-manual occupations were represented by codes 1 (professional), 2 (managerial and technical), 3.1 (non-manual skilled) occupations, while manual occupations were represented by codes 3.2 (manual skilled), 4 (partly skilled), and 5 (unskilled) occupations. Partner's social class was used where available for women and former occupation used where no current occupation was reported [26].

Educational attainment was established using the question “Do you have any of the following qualifications?” followed by a list of common UK qualifications. Participants were categorised according to the highest qualification attained in four groups: those with no formal qualifications; those with formal qualifications usually associated with a school age between 16 ('O' level or equivalent) or 18 years ('A' level or equivalent); and those with degree level qualifications.

Smoking status was derived from two questions each of which could be answered as yes or no: “Have you ever smoked as much as one cigarette a day for as long as a year?” and for those who answered “yes” to the first question “Do you smoke cigarettes now?”

Participants were asked: “Has a doctor ever told you that you have any of the following?” followed by a list of conditions including diabetes, heart attack, stroke and cancer. Personal history of disease was defined by “yes” responses to these four conditions. Trained nurses measured height and weight according to standard protocols at the health examination. Body Mass Index (BMI) was calculated as weight in kilogrammes divided by height squared in square metres.

### Ascertainment of hospital usage and mortality through record linkage

Between 1999 and 2009, cohort participants were also linked to hospital records held by the East Norfolk Primary Health Care Trust using their unique National Health Service number. The database contained hospital episode statistics (HES) coded using the International Classification of Disease (ICD), revision 10, for all Norfolk residents wherever they were treated, including hospitals in other areas in the UK. Linking the EPIC-Norfolk cohort to HES records enables a well defined population denominator to explore future hospital usage patterns. Details of the linkage and outcome variables have been previously reported [22].

Time in hospital over the ten year period was calculated using admission and discharge dates from HES. The sum (in days) of one plus (discharge date minus admission date) was used in order that day cases (admission and discharge on the same day) were considered as well as bed days (overnight stays). The number of hospital admissions was also determined from the HES data with contiguous admissions counted as a single admission. Three dichotomous outcome categories were then calculated: 'Any hospital admissions' and '7 or more admissions' using total admissions and '>20 hospital days' using total bed days and day cases. In addition to total hospital usage for any reason, we also explored usage related to conditions that have been associated with alcohol in systematic literature reviews [8,27].

All participants were followed up for mortality by cause by flagging for death at the UK Office for National Statistics (ONS), and trained nosologists coded death certificates using the ICD, revisions 9 and 10.

### Statistical analysis

For the analyses presented here, we excluded 625 men and women from the baseline cohort who died before 1999 and excluded 1274 for whom alcohol intake was not known or inconsistent leaving 23,740 individuals. Men and women were examined separately recognising the different alcohol consumption patterns and conditions between the sexes. Logistic regression models were used to examine associations between alcohol intake and hospital usage outcome categories for total admissions, and in exploratory analyses for various diagnostic codes. The terms “beneficial” and “detrimental” used in S3 and S4 Tables were defined by Rehm and colleagues in their systematic reviews of disease burden [8,20] and approximated by the lists of ICD version 10 codes shown. Logistic regression was used rather than survival analysis since the outcomes under examination are the total number of admissions and total bed days and day cases occurring over a fixed period of ten years. The numbers of individuals with missing values for covariates were: 51 BMI, 180 smoking status, 466 social class. Logistic regression was also used to examine the risk of death in alcohol drinkers compared to non-alcohol drinkers over the period under examination. Three sensitivity analyses were conducted: using the random forest non-parametric algorithm for multiple imputation; using the value of 1.8 units per glass of wine instead of 1 unit per glass; admissions limited to those after March 2004. All analyses were performed using the R statistical language (R Foundation for Statistical Computing, Vienna, Austria version 3.4.0 with packages knitr, Gmisc, missForest) and Stata statistical software version 14 (Stata Corporation, College Station, Texas, USA).

## Results

Descriptive characteristics of the 10,883 men and 12,857 women by categories of alcohol intake are shown in Table 1 (for men) and Table 2 (for women). Those reporting no current alcohol intake are divided into lifelong abstainers and former drinkers, while those with intake greater than zero are divided into four categories (0,7], (7,14], (14,21] and >21 units per week. Hospital activity is shown in three categories: any hospital admissions; 7 or more admissions and >20 hospital days. Mean and median admissions and bed days/day cases are shown separately for all cohort participants and only those who had attended hospital during the period under examination to enable estimates based only on those attending hospital as well as estimates using a total population denominator. Means and medians calculated using the cohort denominator are lower since they include non-attenders. Men and women currently drinking more than 21 units per week tended to be younger, more likely to be current smokers and more likely to have drunk >21 units per week in their 20s and 30s. Current heavy drinkers (those consuming more than 35 units per week) comprised 448 (4.1%) men and 24 (0.2%) women while 89 men and 1 woman drank heavily in the past but were current non-drinkers.

**Table 1:** Descriptive characteristics by alcohol category for men in the EPIC-Norfolk cohort 1993–1997 and hospital admission 1999–2009.

	All (n = 10,883)	Lifelong abstainer (n = 207 1.9%)	Former drinker (n = 701 6.4%)	(0,7] units per week (n = 4,873 44.8%)	(7,14] units per week (n = 2,346 21.6%)	(14,21] units per week (n = 1,237 11.4%)	>21 units per week (n = 1,519 14.0%)
**Hospital activity, 1999–2009 (n(%))**							
Any hospital admissions	8,025 (73.7)	149 (72.0)	584 (83.3)	3,671 (75.3)	1,700 (72.5)	867 (70.1)	1,054 (69.4)
7 or more admissions	1,688 (15.5)	30 (14.5)	156 (22.3)	783 (16.1)	336 (14.3)	175 (14.1)	208 (13.7)
20 or more hospital nights	2,316 (21.3)	53 (25.6)	229 (32.7)	1,072 (22.0)	452 (19.3)	224 (18.1)	286 (18.8)
**Total hospital days, 1999–2009**							
Mean ±SD, cohort	16.9 ±43.3	17.8 ±38.4	24.9 ±44.3	17.2 ±43.4	15.6 ±40.1	15.0 ±39.2	16.0 ±50.2
Mean ±SD, hospital attenders†	23.0 ±49.0	24.8 ±43.3	29.9 ±46.9	22.8 ±48.7	21.5 ±45.7	21.4 ±45.4	23.1 ±58.9
Median(IQR), cohort	4.0 (0.0–16.0)	6.0 (0.0–21.0)	9.0 (2.0–30.0)	4.0 (1.0–17.0)	3.0 (0.0–15.0)	3.0 (0.0–14.0)	3.0 (0.0–13.0)
Median(IQR), hospital attenders†	9.0 (3.0–25.0)	12.0 (4.0–28.0)	14.0 (4.0–39.0)	9.0 (3.0–25.0)	8.0 (2.0–22.0)	7.0 (2.0–21.0)	8.0 (2.0–23.0)
**Number of admissions, 1999–2009**							
Mean ±SD, cohort	4.2 ±16.2	3.7 ±5.0	5.5 ±17.5	4.1 ±11.7	4.0 ±19.5	4.0 ±19.8	4.0 ±19.9
Mean ±SD, hospital attenders†	5.6 ±18.7	5.1 ±5.3	6.6 ±19.0	5.5 ±13.2	5.5 ±22.7	5.7 ±23.4	5.8 ±23.7
Median(IQR), cohort	2.0 (0.0–5.0)	2.0 (0.0–5.0)	3.0 (1.0–6.0)	2.0 (1.0–5.0)	2.0 (0.0–4.0)	2.0 (0.0–4.0)	2.0 (0.0–4.0)
Median(IQR), hospital attenders†	3.0 (2.0–6.0)	3.0 (2.0–6.0)	4.0 (2.0–7.0)	3.0 (2.0–6.0)	3.0 (1.0–6.0)	3.0 (1.0–5.0)	3.0 (1.0–5.8)
**Alcohol intake, units per week**							
Mean ±SD	10.2 ±11.9	0.0 ±0.0	0.0 ±0.0	3.0 ±2.0	10.5 ±2.0	17.7 ±2.1	33.4 ±13.1
**Age, years**							
Mean ±SD	59.2 ±9.2	63.6 ±8.2	62.1 ±9.3	59.7 ±9.1	58.8 ±9.3	57.9 ±9.2	57.4 ±9.0
**Prevalent disease (n(%))**							
Prevalent heart disease or stroke	691 (6)	11 (5)	65 (9)	347 (7)	146 (6)	53 (4)	69 (5)
Prevalent cancer	398 (4)	10 (5)	32 (5)	168 (3)	85 (4)	49 (4)	54 (4)
Prevalent diabetes	323 (3)	10 (5)	47 (7)	151 (3)	57 (2)	30 (2)	28 (2)
**Smoking status (n(%))**							
Current	1,308 (12)	11 (5)	107 (15)	552 (11)	236 (10)	144 (12)	258 (17)
Former	5,881 (54)	41 (20)	401 (58)	2,449 (51)	1,287 (55)	725 (59)	978 (65)
Never	3,628 (34)	152 (75)	189 (27)	1,836 (38)	812 (35)	363 (29)	276 (18)
**Body mass index, kg/m**^**2**^							
Mean ±SD	26.5 ±3.3	26.5 ±3.2	26.7 ±3.8	26.4 ±3.3	26.3 ±3.1	26.7 ±3.2	27.0 ±3.4
**Level of education (n(%))**							
Low	3,190 (29)	92 (44)	302 (43)	1,632 (33)	555 (24)	284 (23)	325 (21)
‘O’ level or equivalent	948 (9)	17 (8)	48 (7)	394 (8)	216 (9)	118 (10)	155 (10)
‘A’ level or equivalent	5,037 (46)	69 (33)	294 (42)	2,223 (46)	1,123 (48)	577 (47)	751 (49)
Degree	1,708 (16)	29 (14)	57 (8)	624 (13)	452 (19)	258 (21)	288 (19)
**Social class (n(%))**							
Professional (1)	828 (8)	23 (11)	33 (5)	311 (6)	209 (9)	126 (10)	126 (8)
Technical (2)	4,126 (39)	56 (28)	190 (28)	1,641 (34)	964 (42)	566 (46)	709 (48)
Clerical NM (3.1)	1,345 (13)	32 (16)	78 (11)	612 (13)	319 (14)	136 (11)	168 (11)
Clerical M (3.2)	2,697 (25)	35 (17)	220 (32)	1,361 (28)	549 (24)	247 (20)	285 (19)
Semi-skilled (4)	1,404 (13)	45 (22)	129 (19)	715 (15)	226 (10)	117 (10)	172 (12)
Unskilled (5)	305 (3)	11 (5)	35 (5)	153 (3)	47 (2)	30 (2)	29 (2)
**Past alcohol consumption**‡ **(n(%))**							
(0,7] units per week	3,824 (36)	0 (0)	356 (51)	2,487 (51)	644 (27)	195 (16)	142 (9)
(7,14] units per week	2,299 (22)	0 (0)	128 (18)	1,039 (21)	647 (28)	280 (23)	205 (14)
(14,21] units per week	1,547 (15)	0 (0)	68 (10)	535 (11)	433 (18)	273 (22)	238 (16)
>21 units per week	2,981 (28)	0 (0)	149 (21)	792 (16)	619 (26)	488 (39)	933 (61)

^†^ Denominator restricted to cohort participants who attended hospital during the period under examination

^‡^Past alcohol consumption is defined as the higher of units reported consumed aged 20 and aged 30

Round brackets in intervals denote strict inequalities; square brackets denote non-strict inequalities

**Table 2:** Descriptive characteristics by alcohol category for women in the EPIC-Norfolk cohort 1993–1997 and hospital admission 1999–2009.

	All (n = 12,857)	Lifelong abstainer (n = 873 6.8%)	Former drinker (n = 1,086 8.4%)	(0,7] units per week (n = 8,121 63.2%)	(7,14] units per week (n = 1,911 14.9%)	(14,21] units per week (n = 615 4.8%)	>21 units per week (n = 251 2.0%)
**Hospital activity, 1999–2009 (n(%))**							
Any hospital admissions	9,168 (71.3)	691 (79.2)	843 (77.6)	5,769 (71.0)	1,295 (67.8)	405 (65.9)	165 (65.7)
7 or more admissions	1,562 (12.1)	140 (16.0)	203 (18.7)	963 (11.9)	178 (9.3)	57 (9.3)	21 (8.4)
20 or more hospital nights	2,329 (18.1)	257 (29.4)	291 (26.8)	1,412 (17.4)	251 (13.1)	83 (13.5)	35 (13.9)
**Total hospital days, 1999–2009**							
Mean ±SD, cohort	15.2 ±48.9	22.5 ±43.0	23.8 ±64.7	14.3 ±46.3	11.7 ±53.4	12.8 ±46.6	10.9 ±25.9
Mean ±SD, hospital attenders †	21.3 ±56.7	28.5 ±46.5	30.7 ±72.0	20.2 ±53.9	17.2 ±64.2	19.4 ±56.3	16.6 ±30.5
Median(IQR), cohort	3.0 (0.0–13.0)	6.0 (1.0–25.0)	6.0 (1.0–23.0)	3.0 (0.0–12.0)	2.0 (0.0–9.0)	2.0 (0.0–9.0)	2.0 (0.0–10.0)
Median(IQR), hospital attenders†	7.0 (2.0–21.0)	11.0 (3.0–32.0)	11.0 (3.0–33.0)	6.0 (2.0–20.0)	5.0 (2.0–15.0)	6.0 (2.0–16.0)	5.0 (2.0–16.0)
**Number of admissions, 1999–2009**							
Mean ±SD, cohort	3.5 ±16.3	4.1 ±7.6	5.3 ±34.0	3.3 ±8.4	3.3 ±23.5	3.8 ±27.8	2.3 ±3.6
Mean ±SD, hospital attenders†	4.9 ±19.1	5.1 ±8.2	6.9 ±38.5	4.6 ±9.7	4.9 ±28.4	5.8 ±34.2	3.6 ±4.0
Median(IQR), cohort	2.0 (0.0–4.0)	2.0 (1.0–5.0)	2.0 (1.0–5.0)	2.0 (0.0–4.0)	1.0 (0.0–3.0)	1.0 (0.0–3.0)	1.0 (0.0–3.0)
Median(IQR), hospital attenders†	3.0 (1.0–5.0)	3.0 (2.0–6.0)	3.0 (2.0–6.0)	3.0 (1.0–5.0)	2.0 (1.0–4.0)	2.0 (1.0–5.0)	2.0 (1.0–5.0)
**Alcohol intake, units per week**							
Mean ±SD	4.4 ±5.7	0.0 ±0.0	0.0 ±0.0	2.5 ±1.9	10.1 ±2.0	17.0 ±2.2	27.8 ±7.1
**Age, years**							
Mean ±SD	58.5 ±9.2	63.0 ±8.6	60.5 ±9.1	58.2 ±9.1	57.1 ±9.1	57.4 ±9.4	55.4 ±9.2
**Prevalent disease (n(%))**							
Prevalent heart disease or stroke	272 (2)	35 (4)	48 (4)	152 (2)	28 (1)	8 (1)	1 (0)
Prevalent cancer	838 (7)	58 (7)	71 (7)	527 (6)	117 (6)	44 (7)	21 (8)
Prevalent diabetes	186 (1)	26 (3)	33 (3)	107 (1)	15 (1)	2 (0)	3 (1)
**Smoking status (n(%))**							
Current	1,449 (11)	52 (6)	156 (15)	831 (10)	242 (13)	106 (17)	62 (25)
Former	4,152 (33)	110 (13)	375 (35)	2,472 (31)	790 (42)	291 (47)	114 (46)
Never	7,142 (56)	692 (81)	541 (50)	4,753 (59)	865 (46)	217 (35)	74 (30)
**Body mass index, kg/m**^**2**^							
Mean ±SD	26.2 ±4.3	26.8 ±4.7	26.7 ±4.9	26.2 ±4.4	25.6 ±3.8	25.4 ±3.9	25.7 ±3.7
**Level of education (n(%))**							
Low	5,253 (41)	531 (61)	610 (56)	3,371 (42)	534 (28)	149 (24)	58 (23)
‘O’ level or equivalent	1,518 (12)	69 (8)	111 (10)	1,008 (12)	228 (12)	69 (11)	33 (13)
‘A’ level or equivalent	4,658 (36)	219 (25)	303 (28)	2,943 (36)	821 (43)	268 (44)	104 (41)
Degree	1,428 (11)	54 (6)	62 (6)	799 (10)	328 (17)	129 (21)	56 (22)
**Social class (n(%))**							
Professional (1)	830 (7)	35 (4)	37 (4)	486 (6)	171 (9)	65 (11)	36 (15)
Technical (2)	4,475 (36)	237 (28)	268 (25)	2,670 (34)	864 (46)	314 (51)	122 (50)
Clerical NM (3.1)	2,490 (20)	129 (15)	232 (22)	1,626 (20)	361 (19)	105 (17)	37 (15)
Clerical M (3.2)	2,655 (21)	213 (25)	267 (25)	1,772 (22)	301 (16)	71 (12)	31 (13)
Semi-skilled (4)	1,637 (13)	159 (19)	181 (17)	1,079 (14)	152 (8)	52 (9)	14 (6)
Unskilled (5)	482 (4)	63 (8)	71 (7)	310 (4)	29 (2)	4 (1)	5 (2)
**Past alcohol consumption**‡ **(n(%))**							
(0,7] units per week	9,875 (83)	0 (0)	976 (90)	7,185 (90)	1,304 (68)	322 (52)	88 (35)
(7,14] units per week	1,351 (11)	0 (0)	77 (7)	612 (8)	435 (23)	160 (26)	67 (27)
(14,21] units per week	384 (3)	0 (0)	18 (2)	135 (2)	103 (5)	83 (14)	45 (18)
>21 units per week	248 (2)	0 (0)	15 (1)	68 (1)	65 (3)	49 (8)	51 (20)

^†^ Denominator restricted to cohort participants who attended hospital during the period under examination

^‡^Past alcohol consumption is defined as the higher of units reported consumed aged 20 and aged 30

Round brackets in intervals denote strict inequalities; square brackets denote non-strict inequalities

S1 and S2 Tables show age and mean current intake by categories of hospital admissions and bed days/day cases respectively for men and women separately. Admissions are grouped as: zero, 1, 2–3, 4–6 and ≥7 while bed days/day cases are grouped as zero, day case, 1–4, 5–19 and 20+.

Table 3 shows the relationships between dichotomous and grouped alcohol categories and hospital usage for men and women separately. In Table 3, model 1 (age adjusted) and model 2 (multivariable adjusted) compare non-drinkers with current drinkers while model 3 (multivariable adjusted) compares non-drinkers with intake in four bands. Compared to non-drinkers, men who currently drink had a lower risk of spending more than twenty days in hospital with multivariable adjusted OR 0.80 (95% CI 0.68–0.94). Women who currently drink were also less likely to have any hospital admissions multivariable adjusted OR 0.84 (95% CI 0.74–0.95), seven or more admissions OR 0.77 (95% CI 0.66–0.88) or more than twenty hospital days OR 0.70 (95% CI 0.62–0.80). We did not observe a higher risk of hospitalisation at any level of intake including those consuming 21 units or more per week. Table 4 differs from Table 3 by the use of lifelong abstainers as reference category. Compared to lifelong abstainers, men who currently drink had a higher risk of any hospital admissions OR 1.53 (95% CI 1.10–2.13) while in women the association was inverse OR 0.84 (95% CI 0.70–1.01). Men who were former drinkers had a higher risk than lifelong abstainers OR 2.22 (95% CI 1.51–3.28) while former drinking women showed no difference OR 1.01 (95% CI 0.80–1.27). The associations were similar in all categories of intake.

**Table 3:** Age adjusted and multivariable logistic regression of risk factors for any hospital admissions (compared to none), ≥7 hospital admissions (compared to <7 admissions) and >20 days of hospital stay (compared to ≤20 days) from 1999–2009 in 23,740 men and women aged 40–79 years 1993–1997.

	All	n	Any hospital admissions OR (95% CI)	p value	n	Seven or more admissions OR (95% CI)	p value	n	20 or more hospital nights OR (95% CI)	p value
**Men** †										
Current non-drinker	908	733	1	–	186	1	–	282	1	–
Current drinker	9975	7292	0.81 (0.68–0.97)	0.021	1502	0.84 (0.71–1.00)	0.052	2034	0.74 (0.63–0.87)	<0.001
**Men** ‡										
Current non-drinker	908	733	1	–	186	1	–	282	1	–
Current drinker	9975	7292	0.85 (0.71–1.02)	0.083	1502	0.88 (0.73–1.05)	0.162	2034	0.80 (0.68–0.94)	0.008
**Men** ‡										
Current non-drinker	908	733	1	–	186	1	–	282	1	–
(0,7] units per week	4873	3671	0.89 (0.74–1.08)	0.231	783	0.90 (0.74–1.09)	0.266	1072	0.82 (0.69–0.97)	0.024
(7,14] units per week	2346	1700	0.85 (0.69–1.04)	0.106	336	0.85 (0.68–1.04)	0.120	452	0.76 (0.62–0.92)	0.005
(14,21] units per week	1237	867	0.79 (0.64–0.99)	0.037	175	0.88 (0.69–1.12)	0.284	224	0.75 (0.60–0.94)	0.012
>21 units per week	1519	1054	0.78 (0.63–0.96)	0.020	208	0.85 (0.68–1.08)	0.187	286	0.83 (0.67–1.03)	0.095
**Women** †										
Current non-drinker	1959	1534	1	–	343	1	–	548	1	–
Current drinker	10898	7634	0.77 (0.69–0.87)	<0.001	1219	0.69 (0.61–0.79)	<0.001	1781	0.65 (0.57–0.73)	<0.001
**Women** ‡										
Current non-drinker	1959	1534	1	–	343	1	–	548	1	–
Current drinker	10898	7634	0.84 (0.74–0.95)	0.005	1219	0.77 (0.66–0.88)	<0.001	1781	0.70 (0.62–0.80)	<0.001
**Women** ‡										
Current non-drinker	1959	1534	1	–	343	1	–	548	1	–
(0,7] units per week	8121	5769	0.85 (0.75–0.96)	0.010	963	0.79 (0.68–0.91)	0.001	1412	0.73 (0.64–0.83)	<0.001
(7,14] units per week	1911	1295	0.82 (0.70–0.96)	0.012	178	0.69 (0.56–0.85)	<0.001	251	0.61 (0.51–0.73)	<0.001
(14,21] units per week	615	405	0.75 (0.61–0.93)	0.008	57	0.67 (0.49–0.91)	0.011	83	0.61 (0.46–0.80)	<0.001
>21 units per week	251	165	0.79 (0.59–1.07)	0.124	21	0.63 (0.39–1.01)	0.054	35	0.69 (0.46–1.04)	0.078

OR = Odds ratio, CI = Confidence intervals. Comparison group: Current non-drinker

^†^Adjusted for age

^‡^ Adjusted for age, smoking status, education level (low/others), social class (manual/non-manual), body mass index (continuous), prevalent heart disease or stroke, prevalent cancer and prevalent diabetes

Round brackets in intervals denote strict inequalities; square brackets denote non-strict inequalities

**Table 4:** Age adjusted and multivariable logistic regression of risk factors for any hospital admissions (compared to none), ≥7 hospital admissions (compared to <7 admissions) and >20 days of hospital stay (compared to ≤20 days) from 1999–2009 in 23,740 men and women aged 40–79 years 1993–1997.

	All	n	Any hospital admissions OR (95% CI)	p value	n	Seven or more admissions OR (95% CI)	p value	n	20 or more hospital nights OR (95% CI)	p value
**Men** †										
Lifelong abstainer	207	149	1	–	30	1	–	53	1	–
Former drinker	701	584	2.33 (1.60–3.40)	<0.001	156	1.84 (1.20–2.84)	0.006	229	1.63 (1.13–2.36)	0.009
Current drinker	9975	7292	1.52 (1.11–2.10)	0.010	1502	1.37 (0.92–2.03)	0.123	2034	1.08 (0.78–1.51)	0.632
**Men** ‡										
Lifelong abstainer	207	149	1	–	30	1	–	53	1	–
Former drinker	701	584	2.22 (1.51–3.28)	<0.001	156	1.70 (1.08–2.65)	0.021	229	1.47 (1.00–2.16)	0.051
Current drinker	9975	7292	1.53 (1.10–2.13)	0.011	1502	1.34 (0.89–2.02)	0.163	2034	1.08 (0.76–1.52)	0.676
**Men** ‡										
Lifelong abstainer	207	149	1	–	30	1	–	53	1	–
Former drinker	701	584	2.22 (1.51–3.27)	<0.001	156	1.70 (1.08–2.65)	0.021	229	1.47 (1.00–2.16)	0.051
(0,7] units per week	4873	3671	1.61 (1.15–2.24)	0.005	783	1.37 (0.90–2.07)	0.137	1072	1.10 (0.78–1.57)	0.578
(7,14] units per week	2346	1700	1.53 (1.09–2.14)	0.015	336	1.29 (0.84–1.97)	0.241	452	1.02 (0.71–1.46)	0.917
(14,21] units per week	1237	867	1.43 (1.00–2.02)	0.047	175	1.34 (0.86–2.08)	0.197	224	1.01 (0.69–1.48)	0.952
>21 units per week	1519	1054	1.40 (0.99–1.98)	0.056	208	1.30 (0.84–2.02)	0.233	286	1.13 (0.78–1.63)	0.533
**Women** †										
Lifelong abstainer	873	691	1	–	140	1	–	257	1	–
Former drinker	1086	843	1.04 (0.83–1.30)	0.733	203	1.34 (1.06–1.71)	0.016	291	1.06 (0.86–1.31)	0.569
Current drinker	10898	7634	0.79 (0.66–0.94)	0.007	1219	0.82 (0.67–0.99)	0.042	1781	0.67 (0.57–0.79)	<0.001
**Women** ‡										
Lifelong abstainer	873	691	1	–	140	1	–	257	1	–
Former drinker	1086	843	1.01 (0.80–1.27)	0.924	203	1.30 (1.01–1.67)	0.042	291	1.02 (0.82–1.27)	0.884
Current drinker	10898	7634	0.84 (0.70–1.01)	0.063	1219	0.89 (0.72–1.09)	0.263	1781	0.71 (0.60–0.84)	<0.001
**Women** ‡										
Lifelong abstainer	873	691	1	–	140	1	–	257	1	–
Former drinker	1086	843	1.01 (0.80–1.27)	0.932	203	1.30 (1.01–1.67)	0.043	291	1.02 (0.82–1.26)	0.891
(0,7] units per week	8121	5769	0.85 (0.71–1.02)	0.088	963	0.91 (0.74–1.13)	0.397	1412	0.73 (0.62–0.87)	<0.001
(7,14] units per week	1911	1295	0.82 (0.67–1.01)	0.063	178	0.80 (0.62–1.03)	0.089	251	0.62 (0.50–0.77)	<0.001
(14,21] units per week	615	405	0.76 (0.59–0.97)	0.028	57	0.78 (0.55–1.10)	0.156	83	0.61 (0.46–0.83)	0.001
>21 units per week	251	165	0.80 (0.58–1.10)	0.171	21	0.73 (0.44–1.20)	0.215	35	0.70 (0.46–1.07)	0.099

OR = Odds ratio, CI = Confidence intervals. Comparison group: Lifelong abstainer

^†^Adjusted for age

^‡^ Adjusted for age, smoking status, education level (low/others), social class (manual/non-manual), body mass index (continuous), prevalent heart disease or stroke, prevalent cancer and prevalent diabetes

Round brackets in intervals denote strict inequalities; square brackets denote non-strict inequalities

Table 5 displays logistic regression models for the outcome of any hospital admissions comparing non-drinkers with current drinkers in various subgroups. Separate models for men and women are stratified by a dichotomised subgroup: age above or below 65 years; smoking status; BMI above and below 30kg/m2; manual and non-manual social class; low or other education level and prevalent disease (heart disease, cancer or diabetes). Odds ratios (OR) within all strata were in consistent directions with no interaction by age, smoking status or BMI.

**Table 5:** Logistic regression models for any hospital admissions comparing non-drinkers with current drinkers in subgroups in 23,740 men and women aged 40–79 years 1993–1997.

	Men non-drinker (ref)	Men current drinker OR (95% CI)	Women non-drinker (ref)	Women current drinker OR (95% CI)
**By age above and below 65 years**				
Less than 65 years	1	0.89 (0.72–1.11)	1	0.79 (0.68–0.92)
65 years and above	1	0.79 (0.56–1.12)	1	0.94 (0.75–1.19)
**By smoking status**				
Current smoker	1	0.85 (0.70–1.03)	1	0.85 (0.74–0.96)
Non-smoker	1	0.87 (0.52–1.45)	1	0.64 (0.43–0.96)
**By BMI**				
BMI>30	1	0.92 (0.76–1.11)	1	0.81 (0.70–0.92)
BMI≤30	1	0.50 (0.28–0.90)	1	0.91 (0.68–1.22)
**By social class**				
Manual social class	1	0.90 (0.70–1.16)	1	0.78 (0.66–0.92)
Non-manual social class	1	0.81 (0.62–1.05)	1	0.89 (0.74–1.06)
**By education**				
Low education level	1	0.88 (0.71–1.11)	1	0.77 (0.65–0.91)
Other education level	1	0.80 (0.59–1.10)	1	0.89 (0.75–1.07)
**By prevalent disease**				
No reported disease	1	0.82 (0.68–0.99)	1	0.81 (0.72–0.92)
Pre-existing heart disease, cancer or diabetes	1	1.22 (0.55–2.72)	1	0.70 (0.28–1.72)

OR = Odds ratio, CI = Confidence intervals. Comparison group: Current non-drinker. All models adjusted for age, smoking status, education level (low/others), social class (manual/non-manual) and body mass index (continuous) except where a dichotomous adjustment variable was the subgroup being examined

S3 Table shows relationships between dichotomous and grouped alcohol categories and hospital usage but uses modified admission and bed day/day case counts containing only admissions that include discharge codes entirely attributable to alcohol intake or partly attributable and considered “detrimental” (alcohol intake positively associated with disease) according to previous systematic reviews of the literature. S4 Table shows similar relationships for discharge codes considered “beneficial” (alcohol intake inversely associated with disease) [8]. In both sub-classifications, men and women who currently drink have a lower risk of admission compared to non-drinkers.

Sensitivity analyses using 1.8 units per glass of wine instead of 1 unit (S5 Table) [28] and using multiple imputation (S6 Table) gave similar results to those presented in the main tables. A sensitivity analysis (S7 Table) with admissions limited to those after March 2004 gave similar results for women but attenuated results for men. Participants excluded due to missing alcohol intake (n = 1274) were older and predominantly women (73%) with a lower proportion having non-manual social classes and education to age 16 or above.

## Discussion

In this cohort of middle-aged and older men and women, there was no evidence of a higher hospital usage for current alcohol consumers when compared with those who do not currently report drinking alcohol. Participants who consumed alcohol were not observed to have a higher rate of hospital admission or time in hospital over the observation period of ten years. In fact the results indicate lower hospital usage for current compared to current non-drinkers for both men and women for all levels of alcohol consumption and hospital usage before and after adjustment for age and other factors previously documented to relate to hospital usage in this cohort. There are a number of possible explanations for these findings.

### Confounding

The frequency and pattern of alcohol use is strongly related to age, sex, education, social class, obesity, and prevalent ill health, all of which are also related to hospital use so confounding is a major issue. However, multivariable regression models adjusting for all these variables hardly changed the findings. In addition, we stratified by main confounders (Table 5) as well as excluding those with known prevalent heart disease, cancer and diabetes, and the results remained consistent in the subgroups. However, measurement of covariates might not be sufficiently accurate to ensure adequate adjustment and we cannot exclude the possibility of residual confounding with known or other unknown factors associated with both alcohol intake and hospital usage, which could either attenuate or strengthen the associations.

### Bias

Differential follow-up might have occurred if participants had chosen to use private hospitals instead of NHS hospitals and the alcohol consumption of those participants differed from the study population. Participants in higher social class groups might be higher alcohol consumers and also use private healthcare not recorded in NHS hospital statistics. If this occurred it might attenuate some of the inverse associations observed. However, private health care use is minimal in Norfolk and these results do reflect the use of National Health Service hospitals which is the predominant health care system.

Similarly, differential misclassification in hospital use may be explained by early death rates. Participants who died early from alcohol attributable diseases may have lower hospital usage over the period under examination having not used hospital services for the entire period. This is unlikely as over this time period the risk of death was in fact lower in alcohol drinkers compared to non-alcohol drinkers hazard ratio (OR) 0.67 (95% CI 0.57–0.80) for men and OR 0.66 (95% CI 0.57–0.76) for women. A sensitivity analysis excluding hospital admissions prior to 2004 showed attenuated associations for men which might indicate that prevalent illness could lead to a reduction or cessation in alcohol consumption although this was not apparent in women.

Under-reporting of consumption in this study is likely given the known problems in capturing alcohol intake by questionnaire. Self-reported alcohol consumption in surveys suggest much lower consumption than estimates based on alcohol sales data [29–31]. In the 1998 Australian National Drug Strategy Household Survey, reported intake accounted for only 46.5% of known alcohol sales for the preceding 12-month period. When asked to estimate average consumption, there is a tendency to report a figure closer to median than mean consumption with heavy drinking episodes disregarded. There is also a tendency for past alcohol consumption to be remembered less well than more recent consumption. Questions relating to past consumption are insufficiently sensitive to determine periods of abstaining, binge drinking, patterns of consumption or heavy use. Nevertheless, random measurement errors or systematic underreporting of heavy alcohol consumption would only attenuate the findings observed.

Those who enrol in studies, typically in middle age, represent healthy survivors while those worst affected by alcohol misuse may be less likely to participate. Participants who drink moderately may not be representative of moderate drinkers of similar age in the general population due to differing consumption patterns over the life course [32,33]. It has also been suggested that while high levels of alcohol consumption are associated with harm in all socioeconomic groups, there appears to be a disproportionate level of harm for individuals with low socioeconomic status [34,35]. A meta-analyses that controlled for quality-related study characteristics found that moderate drinking had no net mortality benefit compared with lifetime abstention or occasional drinking [36]. However, in a large study of linked electronic UK health records using recruitment at general practice rather than individual level, moderate drinking was associated with a lower risk of several cardiovascular diseases [37].

### Inclusion of former drinkers in the current non-drinkers reference group

The choice of reference group in describing our results may influence interpretation. Non-drinkers comprise heterogeneous subgroups with different characteristics. Former drinkers may have stopped consuming alcohol because of illness, irrespective of whether their illness was caused by drinking. They have been reported to have increased risk for cardiovascular mortality compared to long-term abstainers, a phenomenon described as the “sick-quitter” hypothesis [11]. Lifelong abstainers, ostensibly an ideal reference group having no exposure, may have characteristics that are unusual in the general population [38–40]. Lifelong teetotalism is rare in men (less than 2% of those in the current study) and the reasons for abstaining such as cultural or religious beliefs, may introduce other biases obscuring the results. It has also been noted that there are substantial inconsistencies in self-reports of lifetime abstention. Others have suggested moderate drinkers with no previous history of heavy drinking as a reference group since that is the most commonly observed behaviour and forms the largest group [12,41]. The consumption of alcohol in middle aged men and women tends to decline with age with the largest decline in heavy drinkers but with a reduction across all intake categories.

We opted to use both current non-drinkers and lifelong abstainers as reference groups in the main analyses presented. In the context of hospital usage our objective was to examine the burden on hospital services of cohort participants in relation to current alcohol use rather than pathological processes that may be involved in alcohol and disease associations. To this extent participants' previous history of alcohol consumption was less relevant than the more pragmatic question of their use of services given their current drinking status. However, we have also presented analyses using the alternative reference group of lifetime abstainer in order to explore better this issue. Estimates are less stable given the very small proportion of men who were lifetime non-drinkers. These analyses suggest that in men, the highest hospital usage was observed in former drinkers but with current alcohol drinkers also having higher hospital usage than lifelong abstainers. However, findings in women were not materially different, irrespective of whether lifelong abstainers or former drinkers were used as a reference group. There was no evidence of the “sick quitter” effect found in women affecting the risk of hospitalisation observed in current drinkers.

### Findings in context

These results are somewhat unexpected in the light of current beliefs about alcohol intake in the general population and hospital usage. The many diseases related to the high consumption of alcohol would lead us to expect a positive association between hospital usage and alcohol intake. Mortality rates for liver disease have increased four-fold since 1970 with liver disease the third most common cause of premature death in the UK [9]. Obesity related diseases also have a profound impact on hospital services and since alcohol's energy density is second only to fat, a positive association might be expected. However, cardiovascular disease is a predominant reason for hospital admissions, and an inverse association between alcohol intake and cardiovascular disease has been reported in many epidemiological studies [10,11,14,42]. While causality has not been established, plausible biological mechanisms such as the reduction of plaque deposit in arteries, the reduction of blood clot formation and the dissolving of blood clots[18], have supported the reported beneficial associations for ischaemic heart disease (IHD) and diabetes at moderate levels of alcohol intake. Hospitalisations might reflect the balance between positive and negative health effects of alcohol consumption in a particular study population. Most studies based on hospital cases without a population denominator are unable to assess the potential impact of moderate alcohol consumption if associated with lower hospital use.

### Strengths of the study

Most studies of hospital use only have data on patients who are hospitalised, that is, cases without denominators so are unable to assess overall risk associated with alcohol consumption in the general population. We were able to examine hospital usage over a defined time period in a clearly defined community based population using a prospective cohort design. Use of record linkage with routinely collected hospital admissions data means that ascertainment is virtually complete as use of private healthcare in Norfolk at this time period was minimal. We have previously reported that age, BMI and smoking status predict future hospital use in this cohort over a ten year period of follow-up [22]. Loss to follow-up is small (approximately 2%) as few study participants have moved away from the area they were recruited.

Study participants are very well characterised and we were able to take into account many potentially confounding variables documented to relate to hospital usage in this population as well as prevalent ill health. Income was not measured in EPIC-Norfolk. However, in the UK national health system, income is not a major determinant of hospital admissions, and education and occupational social class are stronger sociodemographic indicators in this respect than income. EPIC-Norfolk is homogeneous with respect to race and ethnicity with 99% describing themselves as white. The assessment by study participants of their alcohol intake in their 20s and 30s enabled us to differentiate between current non-drinkers and lifelong abstainers.

The measurement of past alcohol consumption allows the separation of non-drinkers into former drinkers and lifelong abstainers.

### Limitations in generalisability

Potential selection biases may limit the interpretation of the data since participants were recruited in middle-age and represent survivors who may over-represent resilient and less risky drinkers. Since very few cohort participants reported heavy drinking, a limitation of the study is the inability to examine any possibly deleterious effect of very high consumption. While we did not observe a higher risk of admissions even with the highest alcohol intake categories when comparing current non-drinkers to current drinkers, there were very few people in this study population with very high alcohol consumption levels. Hence the generalisability of these findings to other populations where there are substantially more heavy drinkers may be limited. The use of current non-drinker as reference category must also be considered alongside any interpretation of these results as evidence that the consumption of alcohol may be beneficial but we had very few men who were lifelong abstainers in this cohort.

By using total hospital usage, we were able to assess hospital admissions not just for conditions for which alcohol might increase risk, but also the possible lower service use if alcohol at moderate intake levels were to have the postulated cardioprotective effects. The results presented here reflect hospital usage in a middle aged and older age group and thus we are not able to comment on associations in younger people where binge drinking resulting in acute alcohol poisoning, road traffic and other accidents are a major problem. Nevertheless, older people are by far the greatest users of hospital services and in this older cohort, which was similar to UK national samples in many respects, there was no evidence that current alcohol intake was associated with a higher level of hospital use.

## Conclusions

Current alcohol consumption was not associated with higher but lower hospital usage compared with current non-drinkers in this middle aged and older population. The associations were consistent after multivariable adjustment for age, smoking, BMI, education, social class and prevalent illness in both men and women. In men, this association may in part be due to whether former drinkers are included in the non-drinker reference group but in women, the association was consistent irrespective of the choice of reference group. We should note however, that there were few participants in this cohort with very high current alcohol intake. The measurement of past drinking, the separation of non-drinkers into former drinkers and lifelong abstainers and the choice of reference group are all influential in interpreting the risk of alcohol consumption on future hospitalisation.

## Supporting information

S1 Table Distribution of characteristics of 23,740 men and women in 1993–1997 by category of number of hospital admissions 1999–2009. (PDF)

S2 Table Distribution of characteristics of 23,740 men and women in 1993–1997 by category of total hospital days 1999–2009. (PDF)

S3 Table Age adjusted and multivariable logistic regression of risk factors restricted to “detrimental” hospital admissions for any hospital admissions, ≥7 admissions and >20 days of hospital stay from 1999–2009 in 23,740 men and women aged 40–79 years 1993–1997. (PDF)

S4 Table Age adjusted and multivariable logistic regression of risk factors restricted to “beneficial” hospital admissions for any hospital admissions, ≥7 admissions and >20 days of hospital stay from 1999–2009 in 23,740 men and women aged 40–79 years 1993–1997. (PDF)

S5 Table Sensitivity analysis for wine strength 1.8 units per glass Age adjusted and multivariable logistic regression of risk factors for any hospital admissions, ≥7 hospital admissions and >20 days of hospital stay from 1999–2009 in 23,740 men and women aged 40–79 years 1993–1997. (PDF)

S6 Table Sensitivity analysis using multiple imputation using the random forest non-parametric algorithm Age adjusted and multivariable logistic regression of risk factors for any hospital admissions, ≥7 hospital admissions and >20 days of hospital stay from 1999–2009 in 25,639 men and women aged 40–79 years 1993–1997. (PDF)

S7 Table Sensitivity analysis excluding hospital events before April 2004 Age adjusted and multivariable logistic regression of risk factors for any hospital admissions, ≥7 admissions and >20 days of hospital stay from 2004–2009 in 23,740 men and women aged 40–79 years 1993–1997. (PDF)
