# Supplementary material for: Alcohol consumption and future hospital usage: The EPIC-Norfolk prospective population study
Source: PLoS One. 2018 Jul 18;13(7):e0200747. doi: 10.1371/journal.pone.0200747 (PMC6051641; doi:10.1371/journal.pone.0200747)
Supplement: S1 Table — (PDF) [file pone.0200747.s001.pdf]

S1 Table. Distribution of characteristics of 23,740 men and women in 1993–1997 by category of number of hospital admissions 1999–2009 †

	**0** (n=6,547 27.6%)	**1** (n=4,389 18.5%)	**2–3** (n=5,695 24.0%)	**4–6** (n=3,859 16.3%)	**≥7** (n=3,250 13.7%)
**Alcohol intake, men, units per week**					
Mean ±SD	11.2 ±12.1	10.4 ±11.6	10.1 ±11.9	9.5 ±11.9	9.4 ±11.7
**Alcohol intake, women, units per week**					
Mean ±SD	5.0 ±6.0	4.8 ±5.9	4.2 ±5.6	4.0 ±5.4	3.6 ±5.1
**Age, men, years**					
Mean ±SD	55.0 ±8.5	57.3 ±8.8	60.2 ±9.0	62.5 ±8.7	63.6 ±8.2
**Age, women, years**					
Mean ±SD	55.5 ±8.5	56.9 ±8.9	59.4 ±9.2	61.9 ±9.0	62.0 ±9.0

† Includes day cases where admission and discharge are on the same day
