# Supplementary material for: Alcohol consumption and future hospital usage: The EPIC-Norfolk prospective population study
Source: PLoS One. 2018 Jul 18;13(7):e0200747. doi: 10.1371/journal.pone.0200747 (PMC6051641; doi:10.1371/journal.pone.0200747)
Supplement: S2 Table — (PDF) [file pone.0200747.s002.pdf]

S2 Table. Distribution of characteristics of 23,740 men and women in 1993–1997 by category of total hospital days 1999–2009

	None (n=6,547 27.6%)	Day case (n=2,676 11.3%)	1–4 nights (n=4,707 19.8%)	5–19 nights (n=5,165 21.8%)	≥20 nights (n=4,645 19.6%)
**Alcohol intake, men, units per week**					
Mean ±SD	11.2 ±12.1	10.2 ±11.2	10.5 ±11.9	9.7 ±11.6	9.4 ±12.2
**Alcohol intake, women, units per week**					
Mean ±SD	5.0 ±6.0	4.8 ±5.5	4.5 ±6.0	4.2 ±5.5	3.5 ±5.1
**Age, men, years**					
Mean ±SD	55.0 ±8.5	56.1 ±8.2	58.2 ±8.7	60.9 ±8.7	65.1 ±8.0
**Age, women, years**					
Mean ±SD	55.5 ±8.5	55.5 ±8.6	57.7 ±8.8	59.9 ±8.9	64.4 ±8.5
