# Supplementary material for: Alcohol consumption and future hospital usage: The EPIC-Norfolk prospective population study
Source: PLoS One. 2018 Jul 18;13(7):e0200747. doi: 10.1371/journal.pone.0200747 (PMC6051641; doi:10.1371/journal.pone.0200747)
Supplement: S3 Table — (PDF) [file pone.0200747.s003.pdf]

S3 Table. Age adjusted and multivariable logistic regression of risk factors restricted to "detrimental" hospital admissions (those directly associated to alcohol intake in systematic reviews) for any hospital admissions (compared to none), ≥7 admissions (compared to <7 admissions) and >20 days of hospital stay (compared to ≤20 days) from 1999–2009 in 23,740 men and women aged 40–79 years 1993–1997

	**All**	**n**	**Any hospital admissions OR (95% CI)**	**p value**	**n**	**Seven or more admissions OR (95% CI)**	**p value**	**n**	**20 or more hospital nights OR (95% CI)**	**p value**
**Men †**										
Current non-drinker	908	438	1	–	34	1	–	117	1	–
Current drinker	9975	3752	0.77 (0.67–0.89)	<0.001	298	0.92 (0.64–1.32)	0.642	841	0.82 (0.66–1.01)	0.063
**Men ‡**										
Current non-drinker	908	438	1	–	34	1	–	117	1	–
Current drinker	9975	3752	0.78 (0.67–0.90)	<0.001	298	0.98 (0.67–1.43)	0.926	841	0.85 (0.68–1.06)	0.154
**Men ‡**										
Current non-drinker	908	438	1	–	34	1	–	117	1	–
(0,7] units per week	4873	1891	0.78 (0.67–0.91)	0.001	164	1.05 (0.71–1.55)	0.813	432	0.83 (0.66–1.05)	0.123
(7,14] units per week	2346	855	0.75 (0.64–0.89)	<0.001	69	1.01 (0.65–1.56)	0.966	187	0.82 (0.63–1.07)	0.146
(14,21] units per week	1237	426	0.72 (0.60–0.87)	<0.001	26	0.74 (0.43–1.26)	0.261	92	0.83 (0.61–1.12)	0.221
>21 units per week	1519	580	0.86 (0.72–1.03)	0.099	39	0.87 (0.54–1.43)	0.591	130	1.02 (0.76–1.35)	0.913
**Women †**										
Current non-drinker	1959	937	1	–	65	1	–	248	1	–
Current drinker	10898	3929	0.69 (0.62–0.76)	<0.001	262	0.76 (0.58–1.01)	0.060	718	0.64 (0.54–0.74)	<0.001
**Women ‡**										
Current non-drinker	1959	937	1	–	65	1	–	248	1	–
Current drinker	10898	3929	0.74 (0.67–0.82)	<0.001	262	0.85 (0.64–1.14)	0.281	718	0.70 (0.59–0.83)	<0.001
**Women ‡**										
Current non-drinker	1959	937	1	–	65	1	–	248	1	–
(0,7] units per week	8121	2993	0.75 (0.67–0.83)	<0.001	211	0.89 (0.66–1.20)	0.437	571	0.72 (0.60–0.85)	<0.001
(7,14] units per week	1911	652	0.72 (0.63–0.83)	<0.001	33	0.67 (0.43–1.04)	0.076	91	0.57 (0.44–0.74)	<0.001
(14,21] units per week	615	200	0.67 (0.55–0.82)	<0.001	13	0.80 (0.43–1.49)	0.482	36	0.67 (0.45–0.98)	0.038
>21 units per week	251	84	0.72 (0.54–0.96)	0.024	5	0.75 (0.30–1.92)	0.551	20	1.04 (0.62–1.73)	0.893

OR = Odds ratio, CI = Confidence intervals. Comparison group: Current non-drinker †Adjusted for age ‡ Adjusted for age, smoking status, education level(low/others), social class (manual/non-manual), body mass index (continuous), prevalent heart disease or stroke, prevalent cancer and prevalent diabetes

Round brackets in intervals denote strict inequalities; square brackets denote non-strict inequalities

Restricted to hospital admissions with following ICD-10 codes: A15, A16, A17, A18, A19, B20, B21, B22, B23, B24, B90, C00, C01, C02, C03, C04, C05, C06, C07, C08, C09, C10, C11, C12, C13, C15, C18, C19, C20, C21, C22, C32, C33, C34, C50, D00, D01, D02, D03, D04, D05, D06, D07, D08, D09, D10, D11, D12, D13, D14, D15, D16, D17, D18, D19, D20, D21, D22, D23, D24, D25, D26, D27, D28, D29, D30, D31, D32, D33, D34, D35, D36, D37, D38, D39, D40, D41, D42, D43, D44, D45, D46, D47, D48, E24, F10, F32, F33, F34, G31, G40, G41, G62, G72, I11, I12, I13, I42, I47, I48, I60, I61, I62, I85, J09, J10, J11, J12, J13, J14, J15, J16, J17, J18, J19, J20, J21, J22, J85, K20, K21, K22, K28, K29, K30, K31, K38, K57, K58, K59, K60, K61, K62, K63, K70, K73, K74, K75, K76, K77, K80, K81, K82, K83, K85, K86, K90, K91, K92, L40, L41, O00, O01, O02, O03, O04, O05, O06, O07, O35, O08, P04, P05, P06, P07, P22, P25, P26, P27, P28, Q86, R78, T51, X45, X65, Y15, Y90, V01, V02, V03, V04, V09, V10, V11, V12, V13, V14, V15, V16, V17, V18, V19, V20, V21, V22, V23, V24, V25, V26, V27, V28, V29, V30, V31, V32, V33, V34, V35, V36, V37, V38, V39, V40, V41, V42, V43, V44, V45, V46, V47, V48, V49, V50, V51, V52, V53, V54, V55, V56, V57, V58, V59, V60, V61, V62, V63, V64, V65, V66, V67, V68, V69, V70, V71, V72, V73, V74, V75, V76, V77, V78, V79, V80, V81, V82, V83, V84, V85, V86, V87, V88, V89, V90, V91, V92, V93, V94, V95, V96, V97, V98, V99
