# Supplementary material for: Alcohol consumption and future hospital usage: The EPIC-Norfolk prospective population study
Source: PLoS One. 2018 Jul 18;13(7):e0200747. doi: 10.1371/journal.pone.0200747 (PMC6051641; doi:10.1371/journal.pone.0200747)
Supplement: S4 Table — (PDF) [file pone.0200747.s004.pdf]

S4 Table. Age adjusted and multivariable logistic regression of risk factors restricted to "beneficial" hospital admissions (those inversely associated to alcohol intake in systematic reviews) for any hospital admissions (compared to none), ≥7 admissions (compared to <7 admissions) and >20 days of hospital stay (compared to ≤20 days) from 1999–2009 in 23,740 men and women aged 40–79 years 1993–1997

	All	n	Any hospital admissions OR (95% CI)	p value	n	Seven or more admissions OR (95% CI)	p value	n	20 or more hospital nights OR (95% CI)	p value
**Men †**										
Current non-drinker	908	318	1	–	37	1	–	117	1	–
Current drinker	9975	2433	0.75 (0.64–0.87)	<0.001	212	0.63 (0.44–0.91)	0.013	742	0.73 (0.59–0.90)	0.004
**Men ‡**										
Current non-drinker	908	318	1	–	37	1	–	117	1	–
Current drinker	9975	2433	0.81 (0.69–0.96)	0.013	212	0.77 (0.52–1.13)	0.180	742	0.82 (0.65–1.03)	0.091
**Men ‡**										
Current non-drinker	908	318	1	–	37	1	–	117	1	–
(0,7] units per week	4873	1320	0.88 (0.74–1.04)	0.135	119	0.79 (0.52–1.19)	0.252	408	0.85 (0.66–1.08)	0.177
(7,14] units per week	2346	541	0.78 (0.64–0.94)	0.010	45	0.76 (0.47–1.22)	0.253	170	0.85 (0.64–1.12)	0.239
(14,21] units per week	1237	256	0.70 (0.56–0.87)	0.001	30	0.98 (0.57–1.67)	0.936	79	0.75 (0.54–1.05)	0.094
>21 units per week	1519	316	0.71 (0.58–0.88)	0.002	18	0.49 (0.27–0.91)	0.022	85	0.70 (0.51–0.97)	0.031
**Women †**										
Current non-drinker	1959	525	1	–	32	1	–	180	1	–
Current drinker	10898	1615	0.60 (0.53–0.68)	<0.001	79	0.56 (0.37–0.86)	0.008	428	0.57 (0.47–0.69)	<0.001
**Women ‡**										
Current non-drinker	1959	525	1	–	32	1	–	180	1	–
Current drinker	10898	1615	0.67 (0.59–0.77)	<0.001	79	0.87 (0.55–1.37)	0.543	428	0.66 (0.54–0.81)	<0.001
**Women ‡**										
Current non-drinker	1959	525	1	–	32	1	–	180	1	–
(0,7] units per week	8121	1278	0.69 (0.60–0.78)	<0.001	67	0.89 (0.56–1.42)	0.626	354	0.69 (0.56–0.85)	<0.001
(7,14] units per week	1911	238	0.64 (0.53–0.77)	<0.001	9	0.79 (0.36–1.73)	0.551	45	0.47 (0.33–0.67)	<0.001
(14,21] units per week	615	71	0.58 (0.43–0.77)	<0.001	1	0.30 (0.04–2.23)	0.239	21	0.74 (0.46–1.21)	0.237
>21 units per week	251	28	0.60 (0.39–0.94)	0.026	2	1.38 (0.31–6.17)	0.677	8	0.79 (0.37–1.70)	0.546

OR = Odds ratio, CI = Confidence intervals. Comparison group: Current non-drinker †Adjusted for age ‡ Adjusted for age, smoking status, education level(low/others), social class (manual/non-manual), body mass index (continuous), prevalent heart disease or stroke, prevalent cancer and prevalent diabetes

Round brackets in intervals denote strict inequalities; square brackets denote non-strict inequalities

Restricted to hospital admissions with following ICD-10 codes: E10, E11, E12, E13, F01, F02, F03, G30, G31, I20, I21, I22, I23, I24, I25, I63, I64, I65, I66, I67, K80, K81, K82, K83
