# Supplementary material for: Alcohol consumption and future hospital usage: The EPIC-Norfolk prospective population study
Source: PLoS One. 2018 Jul 18;13(7):e0200747. doi: 10.1371/journal.pone.0200747 (PMC6051641; doi:10.1371/journal.pone.0200747)
Supplement: S5 Table — (PDF) [file pone.0200747.s005.pdf]

S5 Table. Sensitivity analysis for wine strength 1.8 units per glass. Age adjusted and multivariable logistic regression of risk factors for any hospital admissions (compared to none), ≥7 hospital admissions (compared to <7 admissions) and >20 days of hospital stay (compared to ≤20 days) from 1999–2009 in 23,740 men and women aged 40–79 years 1993–1997

	All	n	Any hospital admissions OR (95% CI)	p value	n	Seven or more admissions OR (95% CI)	p value	n	20 or more hospital nights OR (95% CI)	p value
**Men †**										
Current non-drinker	908	733	1	–	186	1	–	282	1	–
Current drinker	9975	7292	0.81 (0.68–0.97)	0.021	1502	0.84 (0.71–1.00)	0.052	2034	0.74 (0.63–0.87)	<0.001
**Men ‡**										
Current non-drinker	908	733	1	–	186	1	–	282	1	–
Current drinker	9975	7292	0.85 (0.71–1.02)	0.083	1502	0.88 (0.73–1.05)	0.162	2034	0.80 (0.68–0.94)	0.008
**Men ‡**										
Current non-drinker	908	733	1	–	186	1	–	282	1	–
(0,7] units per week	4466	3377	0.90 (0.74–1.09)	0.271	728	0.91 (0.75–1.10)	0.313	995	0.83 (0.69–0.98)	0.032
(7,14] units per week	2338	1695	0.83 (0.68–1.01)	0.068	348	0.87 (0.71–1.08)	0.200	458	0.75 (0.62–0.91)	0.004
(14,21] units per week	1321	946	0.86 (0.69–1.07)	0.175	182	0.85 (0.67–1.08)	0.195	240	0.76 (0.61–0.95)	0.016
>21 units per week	1850	1274	0.76 (0.62–0.93)	0.009	244	0.82 (0.65–1.02)	0.077	341	0.81 (0.66–0.99)	0.039
**Women †**										
Current non-drinker	1959	1534	1	–	343	1	–	548	1	–
Current drinker	10898	7634	0.77 (0.69–0.87)	<0.001	1219	0.69 (0.61–0.79)	<0.001	1781	0.65 (0.57–0.73)	<0.001
**Women ‡**										
Current non-drinker	1959	1534	1	–	343	1	–	548	1	–
Current drinker	10898	7634	0.84 (0.74–0.95)	0.005	1219	0.77 (0.66–0.88)	<0.001	1781	0.70 (0.62–0.80)	<0.001
**Women ‡**										
Current non-drinker	1959	1534	1	–	343	1	–	548	1	–
(0,7] units per week	7463	5326	0.85 (0.75–0.96)	0.010	899	0.79 (0.68–0.91)	0.001	1330	0.74 (0.65–0.84)	<0.001
(7,14] units per week	2115	1465	0.88 (0.75–1.02)	0.087	211	0.74 (0.61–0.90)	0.002	288	0.62 (0.52–0.74)	<0.001
(14,21] units per week	843	533	0.69 (0.57–0.83)	<0.001	72	0.63 (0.47–0.83)	0.001	109	0.63 (0.49–0.80)	<0.001
>21 units per week	477	310	0.78 (0.62–0.98)	0.035	37	0.59 (0.41–0.85)	0.005	54	0.56 (0.40–0.77)	<0.001

OR = Odds ratio, CI = Confidence intervals. Comparison group: Lifelong abstainer †Adjusted for age ‡ Adjusted for age, smoking status, education level (low/others), social class (manual/non-manual), body mass index (continuous), prevalent heart disease or stroke, prevalent cancer and prevalent diabetes
Round brackets in intervals denote strict inequalities; square brackets denote non-strict inequalities
