# Supplementary material for: Alcohol consumption and future hospital usage: The EPIC-Norfolk prospective population study
Source: PLoS One. 2018 Jul 18;13(7):e0200747. doi: 10.1371/journal.pone.0200747 (PMC6051641; doi:10.1371/journal.pone.0200747)
Supplement: S6 Table — (PDF) [file pone.0200747.s006.pdf]

S6 Table. Sensitivity analysis using multiple imputation using the random forest non-parametric algorithm. Age adjusted and multivariable logistic regression of risk factors for any hospital admissions (compared to none), ≥7 hospital admissions (compared to <7 admissions) and >20 days of hospital stay (compared to ≤20 days) from 1999–2009 in 25,639 men and women aged 40–79 years 1993–1997

	All	n	Any hospital admissions OR (95% CI)	p value	n	Seven or more admissions OR (95% CI)	p value	n	20 or more hospital nights OR (95% CI)	p value
**Men †**										
Current non-drinker	1091	856	1	–	211	1	–	327	1	–
Current drinker	10516	7575	0.85 (0.73–0.99)	0.039	1562	0.87 (0.74–1.03)	0.097	2146	0.77 (0.66–0.89)	<0.001
**Men ‡**										
Current non-drinker	1091	856	1	–	211	1	–	327	1	–
Current drinker	10516	7575	0.91 (0.78–1.06)	0.236	1562	0.93 (0.79–1.10)	0.393	2146	0.83 (0.71–0.96)	0.013
**Men ‡**										
Current non-drinker	1091	856	1	–	211	1	–	327	1	–
(0,7] units per week	5211	3855	0.95 (0.80–1.11)	0.501	824	0.95 (0.80–1.13)	0.570	1141	0.84 (0.72–0.98)	0.028
(7,14] units per week	2432	1747	0.93 (0.78–1.11)	0.400	345	0.91 (0.75–1.11)	0.335	469	0.80 (0.67–0.95)	0.012
(14,21] units per week	1285	890	0.86 (0.70–1.04)	0.119	181	0.93 (0.74–1.17)	0.553	238	0.81 (0.66–1.00)	0.050
>21 units per week	1588	1083	0.81 (0.67–0.97)	0.025	212	0.88 (0.70–1.09)	0.233	298	0.84 (0.69–1.03)	0.087
**Women †**										
Current non-drinker	2372	1843	1	–	402	1	–	649	1	–
Current drinker	11660	8134	0.77 (0.69–0.86)	<0.001	1313	0.71 (0.63–0.81)	<0.001	1961	0.68 (0.61–0.75)	<0.001
**Women ‡**										
Current non-drinker	2372	1843	1	–	402	1	–	649	1	–
Current drinker	11660	8134	0.85 (0.76–0.94)	0.002	1313	0.79 (0.70–0.90)	<0.001	1961	0.75 (0.67–0.84)	<0.001
**Women ‡**										
Current non-drinker	2372	1843	1	–	402	1	–	649	1	–
(0,7] units per week	8747	6184	0.86 (0.77–0.96)	0.006	1045	0.82 (0.72–0.93)	0.003	1558	0.77 (0.69–0.86)	<0.001
(7,14] units per week	2001	1347	0.82 (0.72–0.95)	0.007	186	0.70 (0.57–0.84)	<0.001	270	0.64 (0.54–0.76)	<0.001
(14,21] units per week	646	429	0.79 (0.65–0.96)	0.018	60	0.68 (0.51–0.92)	0.011	92	0.67 (0.52–0.86)	0.002
>21 units per week	266	174	0.79 (0.60–1.04)	0.096	22	0.60 (0.38–0.96)	0.031	41	0.81 (0.56–1.17)	0.255

OR = Odds ratio, CI = Confidence intervals. Comparison group: Lifelong abstainer †Adjusted for age ‡ Adjusted for age, smoking status, education level(low/others), social class (manual/non-manual), body mass index (continuous), prevalent heart disease or stroke, prevalent cancer and prevalent diabetes

Round brackets in intervals denote strict inequalities; square brackets denote non-strict inequalities

Variables used in the multiple imputation include: age, sex, social class, education level, smoking status, beer, wine, sherry, spirits (at present and at age 20 and 30), physical activity, prevalent disease, hospital admission and hospital days
