# Supplementary material for: Alcohol consumption and future hospital usage: The EPIC-Norfolk prospective population study
Source: PLoS One. 2018 Jul 18;13(7):e0200747. doi: 10.1371/journal.pone.0200747 (PMC6051641; doi:10.1371/journal.pone.0200747)
Supplement: S7 Table — (PDF) [file pone.0200747.s007.pdf]

S7 Table. Sensitivity analysis excluding hospital events before April 2004. Age adjusted and multivariable logistic regression of risk factors for any hospital admissions (compared to none), ≥7 admissions (compared to <7 admissions) and >20 days of hospital stay (compared to ≤20 days) from 2004–2009 in 23,740 men and women aged 40–79 years 1993–1997

	All	n	Any hospital admissions OR (95% CI)	p value	n	Seven or more admissions OR (95% CI)	p value	n	20 or more hospital nights OR (95% CI)	p value
**Men †**										
Current non-drinker	908	490	1	–	58	1	–	118	1	–
Current drinker	9975	5154	1.03 (0.89–1.18)	0.696	550	0.99 (0.75–1.31)	0.948	980	0.95 (0.77–1.17)	0.616
**Men ‡**										
Current non-drinker	908	490	1	–	58	1	–	118	1	–
Current drinker	9975	5154	1.03 (0.90–1.19)	0.648	550	1.01 (0.76–1.35)	0.920	980	0.99 (0.80–1.23)	0.932
**Men ‡**										
Current non-drinker	908	490	1	–	58	1	–	118	1	–
(0,7] units per week	4873	2597	1.07 (0.92–1.24)	0.400	295	1.08 (0.80–1.46)	0.598	512	1.01 (0.81–1.27)	0.918
(7,14] units per week	2346	1215	1.05 (0.89–1.23)	0.579	120	0.94 (0.67–1.31)	0.715	227	0.99 (0.77–1.28)	0.969
(14,21] units per week	1237	621	1.01 (0.85–1.21)	0.895	59	0.91 (0.62–1.33)	0.631	106	0.90 (0.67–1.21)	0.477
>21 units per week	1519	721	0.92 (0.77–1.09)	0.332	76	0.95 (0.66–1.37)	0.795	135	0.97 (0.73–1.29)	0.840
**Women †**										
Current non-drinker	1959	1128	1	–	94	1	–	280	1	–
Current drinker	10898	5361	0.80 (0.73–0.89)	<0.001	429	0.87 (0.69–1.10)	0.250	889	0.69 (0.60–0.80)	<0.001
**Women ‡**										
Current non-drinker	1959	1128	1	–	94	1	–	280	1	–
Current drinker	10898	5361	0.86 (0.77–0.95)	0.004	429	0.99 (0.78–1.27)	0.956	889	0.76 (0.65–0.89)	<0.001
**Women ‡**										
Current non-drinker	1959	1128	1	–	94	1	–	280	1	–
(0,7] units per week	8121	4034	0.85 (0.77–0.95)	0.003	325	0.99 (0.77–1.27)	0.917	682	0.75 (0.64–0.89)	<0.001
(7,14] units per week	1911	917	0.87 (0.76–1.00)	0.050	75	1.07 (0.77–1.49)	0.682	132	0.71 (0.56–0.90)	0.004
(14,21] units per week	615	295	0.88 (0.73–1.07)	0.204	22	0.97 (0.59–1.57)	0.891	55	0.93 (0.67–1.28)	0.658
>21 units per week	251	115	0.83 (0.63–1.10)	0.196	7	0.72 (0.33–1.59)	0.418	20	0.82 (0.49–1.38)	0.455

OR = Odds ratio, CI = Confidence intervals. Comparison group: Lifelong abstainer †Adjusted for age ‡ Adjusted for age, smoking status, education level(low/others), social class (manual/non-manual), body mass index (continuous), prevalent heart disease or stroke, prevalent cancer and prevalent diabetes

Round brackets in intervals denote strict inequalities; square brackets denote non-strict inequalities

Variables used in the multiple imputation include: age, sex, social class, education level, smoking status, beer, wine, sherry, spirits (at present and at age 20 and 30), physical activity, prevalent disease, hospital admission and hospital days
